# Genetic Mutations Associated with Isoniazid Resistance in *Mycobacterium tuberculosis*: A Systematic Review

**DOI:** 10.1371/journal.pone.0119628

**Published:** 2015-03-23

**Authors:** Marva Seifert, Donald Catanzaro, Antonino Catanzaro, Timothy C. Rodwell

**Affiliations:** University of California San Diego, School of Medicine, La Jolla, California, United States of America; St. Petersburg Pasteur Institute, RUSSIAN FEDERATION

## Abstract

**Background:**

Tuberculosis (TB) incidence and mortality are declining worldwide; however, poor detection of drug-resistant disease threatens to reverse current progress toward global TB control. Multiple, rapid molecular diagnostic tests have recently been developed to detect genetic mutations in *Mycobacterium tuberculosis (Mtb)* genes known to confer first-line drug resistance. Their utility, though, depends on the frequency and distribution of the resistance associated mutations in the pathogen population. Mutations associated with rifampicin resistance, one of the two first-line drugs, are well understood and appear to occur in a single gene region in >95% of phenotypically resistant isolates. Mutations associated with isoniazid, the other first-line drug, are more complex and occur in multiple *Mtb* genes.

**Objectives/Methodology:**

A systematic review of all published studies from January 2000 through August 2013 was conducted to quantify the frequency of the most common mutations associated with isoniazid resistance, to describe the frequency at which these mutations co-occur, and to identify the regional differences in the distribution of these mutations. Mutation data from 118 publications were extracted and analyzed for 11,411 *Mtb* isolates from 49 countries.

**Principal Findings/Conclusions:**

Globally, 64% of all observed phenotypic isoniazid resistance was associated with the *kat*G315 mutation. The second most frequently observed mutation, *inhA*-15, was reported among 19% of phenotypically resistant isolates. These two mutations, *katG*315 and *inhA*-15, combined with ten of the most commonly occurring mutations in the *inhA* promoter and the *ahpC-oxyR* intergenic region explain 84% of global phenotypic isoniazid resistance. Regional variation in the frequency of individual mutations may limit the sensitivity of molecular diagnostic tests. Well-designed systematic surveys and whole genome sequencing are needed to identify mutation frequencies in geographic regions where rapid molecular tests are currently being deployed, providing a context for interpretation of test results and the opportunity for improving the next generation of diagnostics.

## Introduction

While tuberculosis (TB) incidence and mortality declined over the past decade, there were still an estimated 8.6 million new cases of TB and 1.3 million deaths attributed to TB worldwide in 2012 [1]. Additionally, progress toward the reduction of TB morbidity and mortality has been largely overshadowed by the increasing prevalence of TB that fails to respond to standard antibiotic treatment. Multidrug resistant TB (MDR-TB), or TB that does not respond to either isoniazid (INH) or rifampicin (RIF), the “backbone” of the current recommended treatment, accounted for approximately 3.6% of all new TB cases and 20.2% of recurring, or previously treated TB cases reported in 2012 [1]. On a local level, incidence of MDR-TB is even more alarming, with individual countries detecting MDR-TB among as many as 28% of new cases and 62% of previously treated cases [2].

Drug resistance arises when mutations, or chromosomal replication errors, occur in genes that encode drug targets or drug metabolism mechanisms and impact the efficacy of anti-tuberculosis therapies [1–7]. Suboptimal treatment and poor adherence to drug therapies contribute to resistance amplification; while lack of detection and subsequent inappropriate treatment increase the risk of direct transmission of drug-resistant *Mtb* [5,8,9]. In 2012 it was estimated that less than 25% of MDR-TB cases were detected worldwide [1].

Low MDR-TB detection rates are due in part to the lack of rapid, accurate, and inexpensive diagnostic tests. The most commonly used method for detection of TB disease—sputum smear microscopy—does not detect drug resistance [1]. Standard drug susceptibility testing (DST), based on mycobacterial culture on solid or liquid media, can take weeks to months to produce results and requires significant biosafety resources, making it unfeasible in many low resource settings [10–12]. Multiple genotypic DST methods based on the detection of mutations that confer resistance have been developed during the past decade, including DNA sequencing, line probe assays, hybridization on DNA chips, single-strand-conformation-polymorphisms, pyrosequencing, and real-time PCR [12–14]. In order for these genotypic methods to accurately identify all resistant *Mtb* phenotypes, the genes and specific mutation locations associated with resistance must be known and included in the diagnostic test. However, global frequency and geographic distribution of INH resistance-conferring *Mtb* mutations have not yet been systematically quantified in the pathogen population [15].

Multiple reviews have identified genes that encode drug targets and have summarized the various mechanisms of resistance to both INH and RIF.[6,16–20] While greater than 95% of RIF resistance is associated with mutations in an 81 base pair section of the *rpoB* gene, INH resistance appears more complex and has been associated with multiple genes, most frequently *katG* and *inhA* [17,20–25]. Current rapid molecular diagnostic tests for INH resistance have focused on the detection of the “canonical” mutations in codon 315 of *katG* and position-15 in the *inhA* promoter region. Previous studies have identified highly variable frequencies of these mutations; with *katG*315 mutations accounting for 42 to 95% and *inhA*-15 mutations accounting for 6 to 43% of phenotypic INH resistance [6,19,20,26,27].

To date no systematic review has assessed the geographic variability of the most common mutations associated with INH resistance. Nor have the frequencies for multiple or co-occurring mutations been aggregated in order to understand the overall proportion of phenotypic INH resistance explained by the existing canonical mutations. It is critically important to understand the frequency and geographic distribution of mutations associated with INH resistance. A failure to account for these variations limits the local effectiveness of molecular diagnostic tools currently available and constrains the development of improved genotypic diagnostic tests [28]. The aims of our systematic review were to quantify the frequency and co-occurrence of the most common mutations associated with phenotypic INH drug resistance and to describe the regional differences in the distribution of these mutations as reported in the published literature.

## Methods

A PubMed search was conducted to identify all English-language peer-reviewed publications that assessed mutations associated with INH resistance. In order to capture publications not aggregated in previous reviews, the search was restricted to studies published between January 1, 2000 and August 31, 2013. PubMed key search terms included: (INH OR isoniazid) AND (resistance or resistant) AND (tuberculosis) AND (mutation OR sequence).

### Publication Selection Criteria

Publications were selected for inclusion if they met the following criteria: 1) presented original data; 2) used clinical strains of *Mtb* (not laboratory strains); 3) described the phenotypic DST method used as reference standard; 4) used DNA sequencing (including pyrosequencing) as a means of characterizing mutations; and 5) included individual level amino acid mutation data. Data from studies evaluating commercial or in-house molecular drug susceptibility tests were included if the results were verified by DNA sequencing.

Our primary goal was to assemble mutation data that was representative of the global distribution of drug resistance phenotypes. Studies were excluded if DNA sequencing was only performed on isolates with discrepancies between phenotypic and genotypic tests, as data from these studies would not be generalizable to the pathogen population. Studies were also excluded if DNA sequencing was performed on non-random isolate subsets or if studies reported sequential sequencing, i.e. sequencing *katG* on all isolates then sequencing *inhA* among isolates without *katG* mutations then sequencing *ahpC*-*oxyR* among isolates without mutations in either *inhA* or *katG*. An exception was made for studies where identifying sequencing results for the initial gene sequenced was easily discernible and in these instances, the sequencing results for only the first gene sequenced were included.

### Data Extraction

The following information was extracted from each publication: primary author, publication year, geographic origin of specimens, year(s) of specimen collection, reference strains, phenotypic drug susceptibility testing method and cutoff, genotypic testing method, gene loci sequenced, primers used for DNA sequencing, and total number of resistant and susceptible isolates tested. Individual isolate mutation information included: location of gene mutation, amino acid and/or nucleotide changes, number of resistant and susceptible isolates with identified mutations, and number of resistant and susceptible isolates tested. Mutations in codon 463 of *katG* were not extracted as previous studies have identified *katG*463 as a genetic marker and not a marker of resistance [29,30]. All data were extracted and compiled using MS Excel software (Microsoft, Redmond, WA).

### Mapping of Primer Sequences and Mutation Locations

In order to accurately assess which gene fragments had been sequenced for each individual isolate, the exact start and end points of the sequenced sections were determined using one of two methods. If primer sequences were included or referenced in the selected studies, sequence endpoint coordinates were identified by entering primer sequences into the NCBI Basic Local Alignment Search Tool (BLAST) and mapping the primers onto the complete *Mtb* H37Rv genome (Accession number NC_000962.3) [31]. If primer sequences were not published, sequenced endpoints coordinates were inferred by using the outermost identified mutations as sequence endpoints. If several primers were included and sequenced fragments overlapped, the final dataset included only the outermost/inclusive primer coordinates. Mutations identified in studies by traditional gene and nucleotide or codon nomenclature were converted to NCBI *Mtb* H37Rv complete genome (Accession number NC_000962.3) coordinates using the conversions described in S1 Table. In short, mutations occurring in the promoter or regulatory regions of the identified genes were mapped by exact nucleotide position and mutations occurring in the coding region of the gene were mapped by the center nucleotide position of the codon. Due to inconsistencies in the nomenclature and reporting of insertions, deletions, and frameshifts, they were excluded from analysis.

### Calculation of Single Mutation Frequencies

As described in 2012 by Georghiou et al., mutation frequencies among phenotypically resistant isolates were calculated by aggregating the number of occurrences of a specific mutation among phenotypically resistant isolates and dividing it by the total number of phenotypically resistant isolates that could have identified that mutation, i.e. all phenotypically resistant isolates that had been sequenced at the location of the specific mutation [32]. Mutation frequencies among phenotypically susceptible isolates were calculated using the same method; the number of occurrences of a specific mutation among phenotypically susceptible isolates was divided by the total number of phenotypically susceptible isolates sequenced at the location of the mutation being assessed. Additionally, mutation frequency data were stratified by WHO region based on specimen origin and regional frequencies were calculated as described above [33].

### Calculation of Cumulative Mutation Frequencies

Studies that reported co-occurring or multiple mutations in phenotypically resistant isolates were further analyzed to assess cumulative frequencies of the most commonly occurring mutations. Cumulative mutation frequencies were calculated first for individual genes, then across multiple genes simultaneously. Isolates were only included in data subsets if they had been sequenced at each of the mutation locations being evaluated. Individual mutation frequencies were reassessed in each data subset for global comparison, then the cumulative frequency for all mutations being evaluated was calculated by aggregating the number of isolates containing any of the mutations being assessed and dividing it by the total number of isolates in the subset.

## Results

Of the 466 publications identified, 348 were excluded as they did not meet inclusion criteria (S1 Fig.). Mutation data, including gene, mutation location, original amino acid and/or nucleotide, and mutated amino acid and/or nucleotide, were extracted for 11,411 *Mtb* isolates described in the selected publications. Study size ranged from 4 to 502 isolates per publication. Seventy-seven percent (8,786) of the isolates were phenotypically resistant to INH. Geographic origin of the isolates was reported for 94% (10,745) of the isolates and included 49 countries (S4 Table). Primers were extracted (>96% of publications included primer information) for the three most commonly inspected genes and their surrounding regions: *furA-katG*, *fabG1-inhA*, and *aphC-oxyR*.

Mutations associated with INH resistance were reported in 15 unique gene regions, the most frequently being *furA-katG* (115 publications), *fabG1-inhA* (72 publications), and *ahpC-oxyR* (24 publications). Mutations were also reported in *efpA*, *fadE24*, *iniA*, *iniB*, *iniC*, *kasA*, *nat*, *ndh*, Rv1772, Rv1592c, Rv0340, and *srmR* genes, however data extracted for these genes were very limited and were excluded from further analysis.

### Single Mutation Frequencies

Global frequencies for all mutations occurring in the *katG* coding region, *inhA* coding region, *inhA* promoter region, and *ahpC-oxyR* intergenic region were evaluated, however only mutations occurring at a cumulative frequency of >0.1% and identified in at least two publications are summarized in Table 1 (for a complete listing of all mutations see S2 Table). Of the 8,416 phenotypically resistant isolates sequenced at *katG*315, 5,400 (64.2%) harbored a mutation in *katG*315 whereas only 2 of 2,462 (<0.1%) phenotypically susceptible isolates harbored a mutation at that location. The remaining mutations identified in *katG* had frequencies of less than 1% among phenotypically resistant isolates. The second most commonly occurring mutation in INH resistant isolates, at position-15 of the *inhA* promoter, was identified in 1,189 of 6,192 (19.2%) phenotypically resistant and 7 of 2,063 (0.3%) phenotypically susceptible isolates. A second mutation in *inhA* promoter region, -8, was identified among 1.3% of phenotypically resistant isolates. Additionally, mutations occurring in the coding region of the *inhA* gene at codon positions 21, 94, and 194 were identified in approximately 1% of the isolates. Mutations in the *ahpC-oxyR* intergenic region reported at positions-10, -46, and -6 occurred in just over 1% of phenotypically resistant isolates. However, the mutation at position-46 of *ahpC-oxyR* intergenic region was also reported in 0.9% of susceptible isolates as well making it a non-specific marker of INH resistance.

**Table 1:** Global frequencies for selected mutations among phenotypically isoniazid resistant and phenotypically isoniazid sensitive isolates.

Gene	Loci	H37Rv Coordinate	Frequency among Resistant specimens (%)	Resistant specimens (n)	Frequency among Susceptible specimens (%)	Susceptible specimens (n)
*katG*	315	2155168	64.2	8416	0.1	2462
	309	2155186	0.5	7967	0.0	2277
	316	2155165	0.4	8199	0.0	2393
	311	2155180	0.3	8181	0.0	2382
	299	2155216	0.3	7456	0.0	2052
	321	2155150	0.2	7875	0.0	2288
	275	2155288	0.2	6713	0.0	2015
	328	2155129	0.2	7825	0.0	2288
	155	2155648	0.2	2105	0.0	538
	110	2155783	0.2	2031	0.0	511
*inhA* Promotor	-15	1673425	19.2	6192	0.3	2063
	-8	1673432	1.3	5955	0.0	2048
	-47	1673393	0.4	5914	0.0	1994
	-17	1673423	0.3	6143	0.0	2063
*inhA*	94	1674482	1.2	2225	0.0	444
	21	1674263	1.1	1685	0.0	434
	194	1674782	1.1	996	0.0	286
	3	1674209	0.5	1713	0.0	434
	258	1674974	0.3	926	0.0	286
	190	1674770	0.3	996	0.0	286
ahpC-oxyR Intergenic Region	-10	2726141	1.3	2321	0.0	536
	-46	2726105	1.2	2321	0.9	536
	-6	2726145	1.1	2321	0.0	536
	-39	2726112	0.8	2321	0.0	536
	-48	2726103	0.7	2424	0.0	576
	-15	2726136	0.6	2321	0.0	536
	-12	2726139	0.5	2321	0.0	536
	-9	2726142	0.5	2321	0.0	536
	-100	2726051	0.4	2321	0.2	536
	-30	2726121	0.3	2321	0.0	536
	-32	2726119	0.3	2321	0.2	536
	-81	2726070	0.2	2321	0.0	536

Four-thousand, five-hundred and five isolates had nucleotide information reported in addition to amino acid information for the *katG*315 mutation (Table 2). The most common nucleotide change for the *katG*315 codon, AGC to ACC (serine to threonine), occurred among 93.4% of isolates. AGC to AAC (serine to asparagine), the second most common nucleotide change, occurred among 3.6% of isolates.

**Table 2:** Amino acid and nucleotide mutation frequency of 4,505 *katG*315 (wild type AGC) isolates.

Amino Acid	Nucleotide	Total Number	%
**Threonine**			**95.3**
	ACC	4208	93.4
	ACA	73	1.6
	ACG	7	0.2
	ACT	7	0.2
**Asparagine**			**3.6**
	AAC	161	3.6
**Isoleucine**			**0.5**
	ATC	23	0.5
**Arginine**			**0.4**
	AGA	8	0.2
	CGC	5	0.1
	AGG	4	0.1
**Glycine**			**0.2**
	GGC	9	0.2
**Total**		**4505**	**100.0**

Mutation data at the country level was sparse, allowing for meaningful aggregation only at the regional level. Aggregating mutation frequencies by WHO regions revealed geographic differences in the individual frequencies for the two most commonly occurring mutations, *katG*315 and *inhA-*15 (Table 3). Among phenotypically INH resistant isolates, South East Asia had the highest frequency (78.4%) and the Western Pacific Region had the lowest frequency (55.5%) of the *katG*315 mutation. Frequencies also varied widely for *inhA*-15 mutation with the Americas region reporting the highest frequency (24.6%) and the Eastern Mediterranean region reporting the lowest frequency (13.0%) of this mutation in phenotypically INH resistant isolates.

**Table 3:** Regional distribution of the individual frequencies for *katG*315 and *inhA*-15.

WHO Region	*katG*315			*inhA*-15		
	Frequency (%)	Resistant w/ mutation	Total Resistant	Frequency (%)	Resistant w/ mutation	Total Resistant
**Africa**	73.5	317	431	17.1	71	415
**Americas**	61.6	939	1525	24.6	262	1067
**E Mediterranean**	64.1	334	521	13.0	25	192
**Europe**	69.4	1652	2379	18.7	332	1771
**South East Asia**	78.4	438	559	13.5	56	416
**Western Pacific**	55.5	1378	2483	19.8	371	1873
**Unknown** ^**a**^	66.0	342	518	15.7	72	458
**Total**	64.3	5400	8416	19.2	1189	6192

^a^ Origin of specimens not identified in publication.

### Cumulative Mutation Frequencies

The cumulative frequencies of multiple or co-occurring mutations associated with INH resistance, were assessed first by individual genes and then in combination across multiple gene regions (Table 4). Eighty-nine publications assessed 6,134 phenotypically resistant isolates for multiple mutations in *katG* inclusive of codon positions 306 to 316. The mutation frequency for *katG*315 in this subset of INH resistant isolates was 66.2%. After the inclusion of mutations at codons 311, 309, and 316 as markers of INH resistance, an additional 9 phenotypically resistant isolates were identified, for a total cumulative frequency of 66.3%. For the *inhA* promoter region, a total of 4,484 isolates from 51 publications were sequenced inclusive of positions -8 to -47. The cumulative frequency of *inhA* -15 in this subset was 19.0%, however unlike *katG*, other *inhA* promoter mutations did not typically co-occur with *inhA* -15. By including mutations at *inhA* -8, *inhA* -47, and *inhA* -17 as markers of resistance, an additional 72 (1.6%) phenotypically resistant isolates were identified. Seventeen publications, consisting of 1,696 phenotypically resistant isolates assessed mutations in the *ahpC*-*oxyR* intergenic region. In this subset no co-occurring mutations in the *ahpC-oxyR* intergenic region were observed. Mutations at positions -48, -39, -15, -12, -10, -9, and -6, of the *ahpC*-*oxyR* intergentic region occurred independently, resulting in a global cumulative frequency of 5.4% for *ahpC*-*oxyR* mutations among phenotypically resistant isolates in this subset.

**Table 4:** Single and cumulative mutation frequencies on *katG*, *inhA*, and *ahpC-oxyR* among data subsets which assessed co-occurring mutations.

Gene Name	Mutation	Codon or Nucleotide Location	Resistant	Cumulative
(number of isolates)	type		w/ Mutation	Frequency (%)
*katG* (n = 6,134)	Single	315	4059	66.2
		309	36	0.6
		316	27	0.4
		311	27	0.4
	Cumulative	315, 309, 316, and/or 311	4068	66.3
*inhA* (n = 4,484)	Single	-15	854	19.0
		-8	46	1.0
		-47	15	0.3
		-17	11	0.2
	Cumulative	-15, -8, -47, and/or -17	926	20.6
*ahpC-oxyR* (n = 1,696)	Single	-10	20	1.2
		-6	25	1.5
		-39	13	0.8
		-48	3	0.2
		-15	13	0.8
		-12	9	0.5
		-9	8	0.5
	Cumulative	-10, -6, -39, -48, -15, -12, and/or -9	91	5.4
*katG* & *inhA* (n = 4,179)	Single	katG315	2723	65.2
		inhA -15	795	19.0
	Cumulative	katG315 and/or inhA -15	3337	79.9
*katG* & *inhA* & *ahpC-oxyR* (n = 1,582)	Single	katG315	1056	66.8
		inhA -15	257	16.2
	Cumulative	katG315 and/or inhA -15	1257	79.5
		katG315 and/or inhA -15, -8, -47, -17	1271	80.3
		katG315 and/or inhA -15, -8, -47, & -17 and/or ahpC -10, -6, -39, -48, -15, -12, & -9	1328	83.9

Forty-six studies, consisting of 4,179 phenotypically resistant isolates, assessed mutations in both the *katG* gene and the *inhA* promoter region, specifically encompassing *katG*315, *inhA* -15, *inhA* -8, and *inhA* -17. Frequencies for *katG*315 and *inhA* -15 in this subset were 65.2% and 19.0% respectively. *KatG*315 and *inhA* -15 mutations co-occurred in 181 isolates, resulting in a combined cumulative frequency for *katG*315 and *inhA* -15 of 79.9%. Only fifteen publications, consisting of 1,582 individual isolates, assessed mutations in *katG* coding region, *inhA* promoter region, and *ahpC-oxyR* intergenic region. In this subset, *katG*315 occurred among 66.8% of phenotypically resistant isolates and *inhA* -15 occurred among 16.2% of phenotypically resistant isolates. With the addition of *inhA* -8, *inhA* -47, and *inhA* -17, cumulative frequency reached 80.3%; and with the addition of the *ahpC* -10, *ahpC* -6, *ahpC* -39, *ahpC* -48, *ahpC* -15, *ahpC* -12, and *ahpC* -9 cumulative frequency reached 83.9%.

## Discussion

Few studies to date have attempted to assess the prevalence of the most commonly occurring “canonical” mutations associated with phenotypic INH resistance among a globally representative set of isolates, making it difficult to compare the global frequencies calculated in this systematic review to previous global estimates. However, a study conducted by Lin et al. using 127 INH resistant isolates from California, a population which is thought to mirror global MDR-TB diversity due to immigration, identified a global frequency of 61% for *katG*315, 23% for *inhA* -15 mutations, and 83% for the cumulative frequency of either mutation, approximating the frequencies of these mutations as quantified in this systematic review [34]. In contrast, Campbell et al., using 212 INH resistant isolates from both WHO and CDC laboratory archives estimated the global frequency of the *katG*315 mutation to be 85%, *inhA* -15 to be 17%, and their cumulative frequency 91%; however, isolates used for that study were selected to provide a diverse set of mutation patterns, and therefore may not accurately represent true global frequencies [35]. Finally, a more recent study conducted by Rodwell et al. using 348 INH resistant isolates from four geographically diverse countries estimated the global frequencies of *katG*315 and *inhA* -15 to be significantly higher at 86% and 34% respectively. The cumulative frequency of both mutations with the addition of *inhA -17* was 96% [36]. However, as with the previously mentioned study, isolates were selected to maximize diversity, potentially affecting the generalizability of the frequency estimates.

Prevalence of drug resistant TB is commonly understood to vary significantly by region, making it reasonable to expect regional variation in the frequencies of specific mutations that drive INH resistance [37,38]. Based on this systematic review, frequencies of specific mutations do in fact appear to vary quite significantly by geographic region. Although the total number of isolates analyzed varied by country and for some countries data was very sparse, we suspect that regional differences in the frequencies of the most common mutations could play a significant role in the performance of molecular-based diagnostic tests in some regions. For example, of the 808 INH resistant isolates sequenced from Japan and Korea, only 43% harbored the *katG*315 mutation, in contrast *katG*315 mutations in the post-soviet states of Belarus, Lithuania, Kazakhstan, Latvia, Moldova, and Russia were identified in 94% of the 751 INH resistant isolates analyzed from those countries. This regional variability of the *katG*315 mutation frequency may contribute to the poor performance of molecular diagnostics for INH resistance detection in Japan [39]. However, due to the lack of large, geographically diverse cross-sectional studies, regional patterns in single mutations should be interpreted with caution.

The frequency patterns of the most common mutations associated with INH resistance appear to differ between individual genes. It is clear that the overwhelming majority (64%) of phenotypic INH resistance among *Mtb* isolates is associated with a single mutation, *katG*315. The dominance of this mutation is hypothesized to be the result of a low or zero fitness cost for this mutation, allowing it to propagate without negative selection pressure [40,41]. Mutations other than *katG*315 in the *katG* gene appear to occur at low (<1%) frequencies and occur overwhelmingly in conjunction with the *katG*315 mutation. Patterns of co-occurring mutations in the *inhA* promoter region appear to differ markedly from co-occurring mutations in the *katG* gene. Although the *inhA* -15 mutation is the dominant (19%) mutation in the *inhA* promoter region, other resistance associated mutations (~1%) in the *inhA* promoter region appear to occur independently of the *inhA* -15 mutation and frequently contribute to the detection of INH resistance. In contrast, no single mutation in the *ahpC*-*oxyR* intergenic region showed an individual frequency above 1.3%; rather multiple mutations (at positions -48, -39, -15, -12, -10, -9, and -6,) in the *ahpC*-*oxyR* intergenic region occurred at a total cumulative frequency of 5.4% among phenotypically resistant isolates.

Equally important to our understanding of the distribution of INH resistance associated mutations is their cumulative frequency across genes. Analysis of the cumulative frequencies of mutations in *katG*, *inhA*, and *ahpC*-*oxyR* was performed only in a small subset of the data (n = 1,582), as few publications sequenced portions of all three genes of interest. In this subset, 80% of the isolates had at least one mutation reported at the following locations: *katG*315, *inhA* -15, *inhA* -8, *inhA* -47, or *inhA* -17. With the addition of mutations occurring at *ahpC* -48, *ahpC* -39, *ahpC* -15, *ahpC* -12, *ahpC* -10, *ahpC* -9, or *ahpC* -6, the percentage of isolates with at least one reported mutation increased to 84%. This approaches the cumulative frequency previously documented by Lin et al. of 88% [42]. The increased detection capacity achieved by including mutations in the *ahpC-oxyR* intergenic region appears to contradict previous studies which have found no significant contribution of INH resistance detection from mutations occurring in the *ahpC-oxyR* intergenic region [18,27,43].

## Limitations

Given the nature of a systematic review, we were unable to obtain data from all geographic regions, thereby introducing a theoretical potential for bias in the global cumulative frequencies calculated. However, given that the total number of isolates reviewed exceeded 11,000, the cumulative frequencies most likely approximate true global frequencies of the most common mutations associated with INH resistance. Additionally, there is a potential for publications to selectively sequence isolates that did not show the most common mutations as detected on rapid molecular tests which may have led to artificially lower cumulative frequencies of the most common mutations. To reduce the potential for this reporting bias, we specifically excluded publications that indicated any non-random selection of isolates for sequencing analysis.

## Conclusions

The performance of molecular-based diagnostic tests for drug resistant TB is intrinsically linked with not only the regional frequencies of mutations being detected, but also the diversity of resistance-conferring mutations being detected by diagnostic tests. Both of these factors influence the maximum sensitivity which rapid molecular tests can be expected to achieve.

Based on the data we evaluated, approximately 80% of global *Mtb* isolates with phenotypic resistance to INH appeared to contain mutations in codon 315 of the *katG* gene or position -15 in the *inhA* promoter. Taken together with other independently occurring mutations in the *inhA* promoter and the *ahpC*-*oxyR* intergenic region that have been previously documented to confer INH resistance, a minimum of 84% of all global *Mtb* isolates with INH resistance phenotypes should be detectable with molecular diagnostics based on analysis of these mutations. Additionally, as these mutations almost never (<0.1%) occurred in INH susceptible isolates, they should have specificities in excess of 99% as markers of phenotypic INH resistance. However, the cumulative frequencies of these mutations do appear to differ by region which could lead to variation in the sensitivity of molecular diagnostics if they are based only on these mutations. Further research, especially in regions with low prevalence of *katG* and *inhA* canonical mutations, is needed to determine if unknown genes or specific mutations account for the unexplained phenotypic INH resistance, or if a more inclusive combination of mutations in previously identified genes such as the *inhA* promoter, *inhA* coding, *ahpC-oxyR* intergenic region, or *katG* gene account for the remaining phenotypic resistance. Regional surveys of the most commonly occurring mutations, including mutations reported in the *ahpC-oxyR* intergentic region should continue to be conducted in geographic regions where rapid molecular tests are being deployed. This would allow for tailoring of molecular tests to specific regions, better interpretation of the molecular tests being used, and improved therapy recommendations.

## Supporting Information

S1 Fig PRISMA Flow Diagram. (DOCX)

S1 PRISMA Checklist PRISMA Checklist. (DOC)

S1 Table Calculations for H37Rv gene coordinate locations. (DOCX)

S2 Table Frequency of reported mutations on *furA-katG*, *fabG1-inhA*, and *aphC-oxyR* gene regions. (DOCX)

S3 Table Drug Susceptibility Testing (DST) Methods Employed in Publications. (DOCX)

S4 Table Details of Studies Included in Review. (DOCX)
