# Supplementary material for: Genetic Mutations Associated with Isoniazid Resistance in Mycobacterium tuberculosis: A Systematic Review
Source: PLoS One. 2015 Mar 23;10(3):e0119628. doi: 10.1371/journal.pone.0119628 (PMC4370653; doi:10.1371/journal.pone.0119628)
Supplement: S1 Fig — (DOCX) [file pone.0119628.s001.docx]

**Supplemental Figure S1. PRISMA Flow Diagram**

164 publications

275 publications

365 publications

450 publications

## Eligibility

## Identification

## Reason for Exclusion

16 publications did not present original data

49 publications did not use clinical strains of Mtb

36 publications did not assess INH resistance

90 publications did not perform phenotypic DST and/or DNA Sequencing

46 publications did not identify specimen specific amino acid mutation data

111 publications used ineligible study designs (review, mechanism, or case-study)

466 Publications Identified though Pubmed

118 Publications met eligibility criteria

466 publications

401 publications

## Included
