# Supplementary material for: Genetic Mutations Associated with Isoniazid Resistance in Mycobacterium tuberculosis: A Systematic Review
Source: PLoS One. 2015 Mar 23;10(3):e0119628. doi: 10.1371/journal.pone.0119628 (PMC4370653; doi:10.1371/journal.pone.0119628)
Supplement: S1 Table — (DOCX) [file pone.0119628.s003.docx]

**S1 Table.** Calculations for H37Rv gene coordinate locations.

| Gene Name | Typical presentation | Calculation |
| --- | --- | --- |
| inhA promoter | (-) nucleotide position | 1673440 + (nucleotide location) |
| inhA gene | (+) codon position | 1674202 – 2 + (3*codon location)^a^ |
| fabG1/mabA promoter | (-) nucleotide position | 1673440 + (nucleotide location) |
| fabG1/mabA gene | (+) codon position | 1673440 – 2 + (3*codon location) |
| ahpC gene | (+) codon position | 2726193 – 2 + (3*codon location |
| ahpC-oxyR intergenic region | (-) nucleotide position | 2726193 + (nucleotide location) |
| oxyR gene | (+) codon position | 2726087 + 2 – (3*codon location) |
| katG gene | (+) codon position | 2156113 – (3*codon location) |
| furA-katG promoter | (-) nucleotide position | 2156149 + (nucleotide location) |
| furA gene | (+) codon position | 2156149 + (3*codon location) |

^a^ Calculated H37Rv coordinate is center nucleotide of codon.
