# Supplementary material for: Genetic Mutations Associated with Isoniazid Resistance in Mycobacterium tuberculosis: A Systematic Review
Source: PLoS One. 2015 Mar 23;10(3):e0119628. doi: 10.1371/journal.pone.0119628 (PMC4370653; doi:10.1371/journal.pone.0119628)
Supplement: S2 Table — (DOCX) [file pone.0119628.s004.docx]

**S2 Table.** Frequency of reported mutations on furA-katG, fabG1-inhA, and aphC-oxyR gene regions.

| Listed Gene | Loci | H37Rv Coordinate | Frequency among Resistant Specimens | Frequency among Susceptible Specimens |
| --- | --- | --- | --- | --- |
| mabA | -147 | 1673293 | 0.05% | 0.00% |
| inhA | -113 | 1673327 | 0.02% | 0.00% |
| inhA | -102 | 1673338 | 0.08% | 0.00% |
| inhA | -67 | 1673373 | 0.02% | 0.00% |
| inhA | -63 | 1673377 | 0.04% | 0.00% |
| inhA | -47 | 1673393 | 0.35% | 0.00% |
| inhA | -34 | 1673406 | 0.05% | 0.00% |
| inhA | -24 | 1673416 | 0.03% | 0.00% |
| inhA | -22 | 1673418 | 0.02% | 0.00% |
| fabG1-inhA | -19 | 1673421 | 0.02% | 0.00% |
| inhA | -17 | 1673423 | 0.34% | 0.05% |
| inhA | -15 | 1673425 | 19.21% | 0.34% |
| inhA | -14 | 1673426 | 0.02% | 0.00% |
| inhA | -12 | 1673428 | 0.02% | 0.00% |
| fabG1-inhA | -9 | 1673431 | 0.02% | 0.00% |
| mabA | -8 | 1673432 | 1.25% | 0.00% |
| mabA | -5 | 1673435 | 0.02% | 0.00% |
| fabG1-inhA | 5 | 1673453 | 0.04% | 0.00% |
| fabG1-inhA | 14 | 1673480 | 0.04% | 0.00% |
| mabA | 21 | 1673501 | 0.02% | 0.00% |
| fabG1-inhA | 203 | 1674047 | 0.12% | 0.00% |
| mabA-inhA | 241 | 1674161 | 0.06% | 0.00% |
| inhA | 3 | 1674209 | 0.53% | 0.00% |
| inhA | 16 | 1674248 | 0.06% | 0.00% |
| inhA | 21 | 1674263 | 1.13% | 0.00% |
| inhA | 25 | 1674275 | 0.12% | 0.00% |
| inhA | 44 | 1674332 | 0.12% | 0.00% |
| inhA | 82 | 1674446 | 0.05% | 0.00% |
| inhA | 93 | 1674479 | 0.05% | 0.00% |
| inhA | 94 | 1674482 | 1.21% | 0.00% |
| inhA | 143 | 1674629 | 0.40% | 0.00% |
| inhA | 168 | 1674704 | 0.40% | 0.00% |
| inhA | 181 | 1674743 | 0.20% | 0.00% |
| inhA | 190 | 1674770 | 0.30% | 0.00% |
| inhA | 194 | 1674782 | 1.10% | 0.00% |
| inhA | 203 | 1674809 | 0.40% | 0.00% |
| inhA | 209 | 1674827 | 0.60% | 0.00% |
| inhA | 216 | 1674848 | 0.10% | 0.00% |
| inhA | 235 | 1674905 | 0.40% | 0.00% |
| inhA | 241 | 1674923 | 0.10% | 0.00% |
| inhA | 253 | 1674959 | 0.00% | 0.35% |
| inhA | 256 | 1674968 | 0.11% | 0.35% |
| inhA | 258 | 1674974 | 0.32% | 0.00% |
| inhA | 259 | 1674977 | 0.11% | 0.00% |
| **katG** | | | | |
| katG | 735 | 2153908 | 0.05% | 0.00% |
| katG | 728 | 2153929 | 0.10% | 0.00% |
| katG | 727 | 2153932 | 0.05% | 0.00% |
| katG | 726 | 2153935 | 0.05% | 0.00% |
| katG | 725 | 2153938 | 0.00% | 0.19% |
| katG | 708 | 2153989 | 0.05% | 0.00% |
| katG | 685 | 2154058 | 0.05% | 0.00% |
| katG | 678 | 2154079 | 0.05% | 0.00% |
| katG | 662 | 2154127 | 0.05% | 0.00% |
| katG | 653 | 2154154 | 0.05% | 0.19% |
| katG | 636 | 2154205 | 0.05% | 0.00% |
| katG | 611 | 2154280 | 0.05% | 0.00% |
| katG | 607 | 2154292 | 0.05% | 0.00% |
| katG | 592 | 2154337 | 0.05% | 0.00% |
| katG | 573 | 2154394 | 0.05% | 0.00% |
| katG | 560 | 2154433 | 0.05% | 0.00% |
| katG | 550 | 2154463 | 0.05% | 0.00% |
| katG | 542 | 2154487 | 0.05% | 0.00% |
| katG | 541 | 2154490 | 0.00% | 0.17% |
| katG | 534 | 2154511 | 0.05% | 0.00% |
| katG | 529 | 2154526 | 0.04% | 0.00% |
| katG | 515 | 2154568 | 0.04% | 0.00% |
| katG | 512 | 2154577 | 0.04% | 0.00% |
| katG | 498 | 2154619 | 0.10% | 0.00% |
| katG | 496 | 2154625 | 0.03% | 0.00% |
| katG | 493 | 2154634 | 0.03% | 0.00% |
| katG | 491 | 2154640 | 0.10% | 0.00% |
| katG | 490 | 2154643 | 0.03% | 0.00% |
| katG | 485 | 2154658 | 0.03% | 0.00% |
| katG | 473 | 2154694 | 0.03% | 0.00% |
| katG | 471 | 2154700 | 0.03% | 0.00% |
| katG | 464 | 2154721 | 0.03% | 0.00% |
| katG | 463 | 2154724 | 0.03% | 0.00% |
| katG | 461 | 2154730 | 0.03% | 0.00% |
| katG | 459 | 2154736 | 0.03% | 0.00% |
| katG | 458 | 2154739 | 0.03% | 0.00% |
| katG | 457 | 2154742 | 0.06% | 0.00% |
| katG | 454 | 2154751 | 0.09% | 0.00% |
| katG | 449 | 2154766 | 0.03% | 0.00% |
| katG | 439 | 2154796 | 0.03% | 0.00% |
| katG | 438 | 2154799 | 0.03% | 0.00% |
| katG | 435 | 2154808 | 0.03% | 0.00% |
| katG | 434 | 2154811 | 0.03% | 0.00% |
| katG | 428 | 2154829 | 0.06% | 0.00% |
| katG | 419 | 2154856 | 0.14% | 0.00% |
| katG | 418 | 2154859 | 0.03% | 0.00% |
| katG | 414 | 2154871 | 0.06% | 0.00% |
| katG | 412 | 2154877 | 0.03% | 0.00% |
| katG | 409 | 2154886 | 0.11% | 0.00% |
| katG | 399 | 2154916 | 0.06% | 0.00% |
| katG | 397 | 2154922 | 0.03% | 0.00% |
| katG | 394 | 2154931 | 0.06% | 0.00% |
| katG | 388 | 2154949 | 0.06% | 0.00% |
| katG | 384 | 2154961 | 0.03% | 0.00% |
| katG | 381 | 2154970 | 0.02% | 0.00% |
| katG | 380 | 2154973 | 0.09% | 0.00% |
| katG | 379 | 2154976 | 0.05% | 0.00% |
| katG | 374 | 2154991 | 0.00% | 0.07% |
| katG | 371 | 2155000 | 0.02% | 0.00% |
| katG | 368 | 2155009 | 0.00% | 0.07% |
| katG | 365 | 2155018 | 0.02% | 0.13% |
| katG | 363 | 2155024 | 0.02% | 0.00% |
| katG | 361 | 2155030 | 0.02% | 0.00% |
| katG | 357 | 2155042 | 0.08% | 0.00% |
| katG | 352 | 2155057 | 0.02% | 0.00% |
| katG | 348 | 2155069 | 0.02% | 0.00% |
| katG | 347 | 2155072 | 0.00% | 0.05% |
| katG | 345 | 2155078 | 0.03% | 0.00% |
| katG | 344 | 2155081 | 0.03% | 0.00% |
| katG | 341 | 2155090 | 0.05% | 0.00% |
| katG | 339 | 2155096 | 0.02% | 0.00% |
| katG | 337 | 2155102 | 0.06% | 0.00% |
| katG | 336 | 2155105 | 0.06% | 0.00% |
| katG | 335 | 2155108 | 0.10% | 0.05% |
| katG | 331 | 2155120 | 0.01% | 0.00% |
| katG | 330 | 2155123 | 0.01% | 0.00% |
| katG | 329 | 2155126 | 0.05% | 0.00% |
| katG | 328 | 2155129 | 0.17% | 0.00% |
| katG | 327 | 2155132 | 0.09% | 0.00% |
| katG | 326 | 2155135 | 0.05% | 0.00% |
| katG | 324 | 2155141 | 0.05% | 0.00% |
| katG | 323 | 2155144 | 0.01% | 0.00% |
| katG | 322 | 2155147 | 0.09% | 0.00% |
| katG | 321 | 2155150 | 0.19% | 0.04% |
| katG | 318 | 2155159 | 0.04% | 0.04% |
| katG | 317 | 2155162 | 0.05% | 0.00% |
| katG | 316 | 2155165 | 0.41% | 0.00% |
| katG | 315 | 2155168 | 64.16% | 0.08% |
| katG | 314 | 2155171 | 0.10% | 0.00% |
| katG | 312 | 2155177 | 0.04% | 0.04% |
| katG | 311 | 2155180 | 0.34% | 0.04% |
| katG | 309 | 2155186 | 0.48% | 0.00% |
| katG | 308 | 2155189 | 0.01% | 0.00% |
| katG | 307 | 2155192 | 0.10% | 0.00% |
| katG | 306 | 2155195 | 0.04% | 0.00% |
| katG | 305 | 2155198 | 0.08% | 0.00% |
| katG | 304 | 2155201 | 0.03% | 0.00% |
| katG | 302 | 2155207 | 0.03% | 0.00% |
| katG | 300 | 2155213 | 0.07% | 0.00% |
| katG | 299 | 2155216 | 0.25% | 0.00% |
| katG | 297 | 2155222 | 0.05% | 0.00% |
| katG | 296 | 2155225 | 0.03% | 0.00% |
| katG | 295 | 2155228 | 0.07% | 0.00% |
| katG | 293 | 2155234 | 0.03% | 0.00% |
| katG | 291 | 2155240 | 0.07% | 0.00% |
| katG | 289 | 2155246 | 0.03% | 0.00% |
| katG | 286 | 2155255 | 0.01% | 0.00% |
| katG | 285 | 2155258 | 0.09% | 0.00% |
| katG | 283 | 2155264 | 0.01% | 0.00% |
| katG | 281 | 2155270 | 0.01% | 0.00% |
| katG | 280 | 2155273 | 0.04% | 0.05% |
| katG | 279 | 2155276 | 0.10% | 0.05% |
| katG | 276 | 2155285 | 0.06% | 0.00% |
| katG | 275 | 2155288 | 0.19% | 0.00% |
| katG | 274 | 2155291 | 0.03% | 0.00% |
| katG | 273 | 2155294 | 0.03% | 0.00% |
| katG | 271 | 2155300 | 0.10% | 0.00% |
| katG | 269 | 2155306 | 0.03% | 0.00% |
| katG | 264 | 2155321 | 0.05% | 0.05% |
| katG | 262 | 2155327 | 0.02% | 0.00% |
| katG | 261 | 2155330 | 0.05% | 0.00% |
| katG | 259 | 2155336 | 0.02% | 0.00% |
| katG | 258 | 2155339 | 0.02% | 0.00% |
| katG | 257 | 2155342 | 0.06% | 0.00% |
| katG | 256 | 2155345 | 0.02% | 0.00% |
| katG | 254 | 2155351 | 0.07% | 0.00% |
| katG | 251 | 2155360 | 0.07% | 0.00% |
| katG | 249 | 2155366 | 0.05% | 0.00% |
| katG | 245 | 2155378 | 0.03% | 0.09% |
| katG | 244 | 2155381 | 0.03% | 0.00% |
| katG | 243 | 2155384 | 0.03% | 0.00% |
| katG | 242 | 2155387 | 0.06% | 0.00% |
| katG | 241 | 2155390 | 0.03% | 0.00% |
| katG | 236 | 2155405 | 0.03% | 0.00% |
| katG | 234 | 2155411 | 0.09% | 0.00% |
| katG | 233 | 2155414 | 0.12% | 0.00% |
| katG | 232 | 2155417 | 0.06% | 0.00% |
| katG | 230 | 2155423 | 0.03% | 0.00% |
| katG | 229 | 2155426 | 0.03% | 0.00% |
| katG | 218 | 2155459 | 0.15% | 0.00% |
| katG | 217 | 2155462 | 0.11% | 0.00% |
| katG | 204 | 2155501 | 0.04% | 0.00% |
| katG | 200 | 2155513 | 0.09% | 0.00% |
| katG | 198 | 2155519 | 0.04% | 0.00% |
| katG | 195 | 2155528 | 0.04% | 0.00% |
| katG | 194 | 2155531 | 0.04% | 0.00% |
| katG | 191 | 2155540 | 0.09% | 0.18% |
| katG | 189 | 2155546 | 0.05% | 0.00% |
| katG | 186 | 2155555 | 0.09% | 0.00% |
| katG | 176 | 2155585 | 0.14% | 0.00% |
| katG | 172 | 2155597 | 0.09% | 0.00% |
| katG | 167 | 2155612 | 0.05% | 0.00% |
| katG | 162 | 2155627 | 0.05% | 0.00% |
| katG | 155 | 2155648 | 0.24% | 0.00% |
| katG | 146 | 2155675 | 0.05% | 0.00% |
| katG | 144 | 2155681 | 0.05% | 0.00% |
| katG | 143 | 2155684 | 0.05% | 0.00% |
| katG | 141 | 2155690 | 0.10% | 0.00% |
| katG | 138 | 2155699 | 0.14% | 0.19% |
| katG | 131 | 2155720 | 0.10% | 0.00% |
| katG | 128 | 2155729 | 0.10% | 0.00% |
| katG | 127 | 2155732 | 0.05% | 0.00% |
| katG | 126 | 2155735 | 0.05% | 0.00% |
| katG | 125 | 2155738 | 0.05% | 0.00% |
| katG | 123 | 2155744 | 0.05% | 0.00% |
| katG | 121 | 2155750 | 0.05% | 0.00% |
| katG | 117 | 2155762 | 0.05% | 0.00% |
| katG | 110 | 2155783 | 0.25% | 0.00% |
| katG | 109 | 2155786 | 0.10% | 0.00% |
| katG | 107 | 2155792 | 0.05% | 0.00% |
| katG | 106 | 2155795 | 0.05% | 0.00% |
| katG | 105 | 2155798 | 0.05% | 0.00% |
| katG | 104 | 2155801 | 0.05% | 0.00% |
| katG | 101 | 2155810 | 0.05% | 0.00% |
| katG | 97 | 2155822 | 0.10% | 0.00% |
| katG | 94 | 2155831 | 0.10% | 0.00% |
| katG | 93 | 2155834 | 0.05% | 0.00% |
| katG | 91 | 2155840 | 0.15% | 0.00% |
| katG | 90 | 2155843 | 0.10% | 0.00% |
| katG | 85 | 2155858 | 0.05% | 0.00% |
| katG | 84 | 2155861 | 0.05% | 0.00% |
| katG | 83 | 2155864 | 0.00% | 0.39% |
| katG | 74 | 2155891 | 0.05% | 0.20% |
| katG | 73 | 2155894 | 0.05% | 0.00% |
| katG | 71 | 2155900 | 0.05% | 0.00% |
| katG | 68 | 2155909 | 0.05% | 0.00% |
| katG | 65 | 2155918 | 0.00% | 0.20% |
| katG | 64 | 2155921 | 0.05% | 0.00% |
| katG | 61 | 2155930 | 0.05% | 0.00% |
| katG | 48 | 2155969 | 0.05% | 0.00% |
| katG | 41 | 2155990 | 0.86% | 0.00% |
| katG | 35 | 2156008 | 0.10% | 0.00% |
| katG | 34 | 2156011 | 0.05% | 0.00% |
| katG | 17 | 2156062 | 0.05% | 0.00% |
| katG | 12 | 2156077 | 0.05% | 0.00% |
| katG | 11 | 2156080 | 0.05% | 0.00% |
| katG | 5 | 2156098 | 0.14% | 0.00% |
| katG | 1 | 2156110 | 0.10% | 0.00% |
| katG | -7 | 2156134 | 0.18% | 0.00% |
| katG | -10 | 2156143 | 0.11% | 0.00% |
| katG | -12 | 2156149 | 0.11% | 0.00% |
| katG | -30 | 2156203 | 0.14% | 0.47% |
| **ahpC-oxyR** | | | | |
| oxyR | 13 | 2726050 | 1.42% | 0.00% |
| ahpC-oxyR | -100 | 2726051 | 0.43% | 0.19% |
| ahpC-oxyR | -89 | 2726062 | 0.09% | 0.00% |
| ahpC-oxyR | -88 | 2726063 | 0.39% | 0.00% |
| ahpC-oxyR | -81 | 2726070 | 0.17% | 0.00% |
| ahpC-oxyR | -74 | 2726077 | 0.04% | 0.00% |
| ahpC-oxyR | -57 | 2726094 | 0.04% | 0.00% |
| ahpC-oxyR | -54 | 2726097 | 0.04% | 0.00% |
| ahpC-oxyR | -52 | 2726099 | 0.12% | 0.00% |
| ahpC-oxyR | -49 | 2726102 | 0.04% | 0.00% |
| ahpC-oxyR | -48 | 2726103 | 0.70% | 0.00% |
| ahpC-oxyR | -46 | 2726105 | 1.16% | 0.93% |
| ahpC-oxyR | -40 | 2726111 | 0.09% | 0.00% |
| ahpC-oxyR | -39 | 2726112 | 0.82% | 0.00% |
| ahpC-oxyR | -34 | 2726117 | 0.13% | 0.00% |
| ahpC-oxyR | -32 | 2726119 | 0.26% | 0.19% |
| ahpC-oxyR | -30 | 2726121 | 0.30% | 0.00% |
| ahpC-oxyR | -15 | 2726136 | 0.60% | 0.00% |
| ahpC-oxyR | -12 | 2726139 | 0.52% | 0.00% |
| ahpC-oxyR | -10 | 2726141 | 1.34% | 0.00% |
| ahpC-oxyR | -9 | 2726142 | 0.52% | 0.00% |
| ahpC-oxyR | -6 | 2726145 | 1.08% | 0.00% |
| ahpC-oxyR | 0 | 2726151 | 0.04% | 0.00% |
| ahpC-oxyR | 2 | 2726197 | 0.04% | 0.00% |
| ahpC-oxyR | 3 | 2726200 | 0.04% | 0.00% |
| ahpC-oxyR | 4 | 2726203 | 0.04% | 0.00% |
| ahpC-oxyR | 10 | 2726221 | 0.12% | 0.87% |
| ahpC-oxyR | 20 | 2726251 | 0.14% | 0.00% |
| ahpC-oxyR | 38 | 2726305 | 0.15% | 0.00% |
| ahpC-oxyR | 76 | 2726419 | 0.26% | 0.00% |
| ahpC-oxyR | 92 | 2726467 | 0.26% | 0.00% |
| ahpC-oxyR | 148 | 2726635 | 0.59% | 0.00% |
