# Supplementary material for: Genetic Mutations Associated with Isoniazid Resistance in Mycobacterium tuberculosis: A Systematic Review
Source: PLoS One. 2015 Mar 23;10(3):e0119628. doi: 10.1371/journal.pone.0119628 (PMC4370653; doi:10.1371/journal.pone.0119628)
Supplement: S3 Table — (DOCX) [file pone.0119628.s005.docx]

**S3 Table.** Drug Susceptibility Testing (DST) Methods Employed in Publications.

| Reported DST Method | Critical Concentration (ug/ml) | Number of Articles |
| --- | --- | --- |
| Absolute Concentration |  |  |
| Absolute Concentration | 1.0 | 6 |
| Absolute Concentration | 0.2 | 4 |
| Absolute method – MIC | 1.0 | 1 |
| Absolute method – MIC | 0.5 | 1 |
|  |  |  |
| Radiometric/Liquid media |  |  |
| BACTEC 460/MGIT 960 | 0.1 | 31 |
| BACTEC 460/MGIT 960 | 0.2 | 1 |
| BACTEC 460/MGIT 960 | 1.0 | 1 |
| BACTEC 460/MGIT 960 | Manufacturer Protocol | 14 |
|  |  |  |
|  |  |  |
| Proportion |  |  |
| Proportion Method | 0.2 | 37 |
| Proportion Method | 1 | 2 |
| Proportion Method | Standard Methods* | 10 |
|  |  |  |
| Resistance Ratio Method |  |  |
| Resistance Ratio | 0.2 | 2 |
|  |  |  |
| Other |  |  |
| CDC Guidelines /National Standards | 0.2 | 3 |
| Conventional Culture | Unspecified | 2 |
| CDC Guidelines/WHO Protocol | Standard Methods* | 2 |
| NR (pending) | NR | 1 |

*Performed per Canetti’s Method Protocol, Country of Origin Standards, CDC Guidelines, or WHO Protocol.
