# Supplementary material for: Genetic Mutations Associated with Isoniazid Resistance in Mycobacterium tuberculosis: A Systematic Review
Source: PLoS One. 2015 Mar 23;10(3):e0119628. doi: 10.1371/journal.pone.0119628 (PMC4370653; doi:10.1371/journal.pone.0119628)
Supplement: S4 Table — (DOCX) [file pone.0119628.s006.docx]

**S4 Table.** Details of Studies Included in Review.

| PMID | First Author | Year of Publication | WHO Region | Final Year of Specimen Collection | Total Specimens Extracted* |
| --- | --- | --- | --- | --- | --- |
| **10897383** | Kiepiela | 2000 | Africa | 1995 | 96 |
| **10970356** | Torres | 2000 | WW | NR | 56 |
| **11179917** | Harris | 2000 | Western Pacific | 1996 | 29 |
| **11408244** | Lee | 2001 | Western Pacific | 1996 | 106 |
| **11453593** | Abate | 2001 | WW | NR | 42 |
| **11796356** | Siddiqi | 2002 | South East Asia | 1998 | 73 |
| **11931416** | Torres | 2002 | Europe | 1999 | 66 |
| **12089271** | Mokrousov | 2002 | Europe | 2001 | 6 |
| **12354882** | Tracevska | 2002 | Europe | 2000 | 51 |
| **12365580** | Ahmad | 2002 | Eastern Mediterranean | 2000 | 58 |
| **12654653** | Ramaswamy | 2003 | Americas | NR | 124 |
| **12663163** | Torres | 2002 | Europe | NR | 43 |
| **12760887** | Bakonyte | 2003 | Europe | 2002 | 235 |
| **12958298** | Silva | 2003 | Americas | 1999 | 69 |
| **14532194** | Van Doorn | 2003 | Europe | NR | 20 |
| **14532345** | Leung | 2003 | Western Pacific | 2002 | 20 |
| **14596968** | Kim | 2003 | Western Pacific | 2002 | 97 |
| **14638486** | Morlock | 2003 | Americas and Europe | NR | 41 |
| **14729930** | Ramaswamy | 2004 | Americas | 1998 | 50 |
| **15184414** | Sajduda | 2004 | Europe | 2000 | 87 |
| **15194127** | Herrera | 2004 | Western Pacific | 2002 | 153 |
| **15328099** | Cardoso | 2004 | Americas | 2001 | 157 |
| **15527151** | Shemyakin | 2004 | Europe | 2001 | 96 |
| **15616288** | Herrera-Leon | 2005 | Europe | 2003 | 347 |
| **15675556** | Hofling | 2005 | Americas | 2000 | 48 |
| **15675557** | Rindi | 2005 | Europe | 2002 | 45 |
| **15679486** | Arnold | 2005 | Europe | NR | 97 |
| **15786899** | Zhao | 2005 | Western Pacific | NR | 42 |
| **15879493** | Espasa | 2005 | Europe | 2001 | 53 |
| **15910221** | Aktas | 2005 | Europe | 2002 | 56 |
| **15910223** | Coll | 2005 | Europe | 1997 | 53 |
| **15917515** | Parsons | 2005 | Americas | 1994 | 157 |
| **16081898** | Hillemann | 2005 | Europe | 2001 | 143 |
| **16189082** | Lavender | 2005 | Western Pacific | 2003 | 104 |
| **16243477** | Yang | 2005 | Europe | NR | 95 |
| **16249627** | Ozturk | 2005 | Europe | NR | 4 |
| **16272473** | Zhang | 2005 | Western Pacific | NR | 137 |
| **16458476** | Kim | 2006 | Western Pacific | 2002 | 96 |
| **16672384** | Park | 2006 | Western Pacific | NR | 243 |
| **16789833** | Gagneux | 2006 | Americas | 1999 | 136 |
| **16825346** | Cavusoglu | 2006 | Europe | 2004 | 37 |
| **16825369** | Miotto | 2006 | Europe | 2005 | 206 |
| **16872043** | Negi | 2006 | South East Asia | 2004 | 6 |
| **16928967** | Marais | 2006 | Africa | 3005 | 49 |
| **17030912** | Guo | 2006 | Americas |  | 28 |
| **17035488** | Somoskovi | 2006 | Americas | 2005 | 143 |
| **17108078** | Sekiguchi | 2007 | Western Pacific and Europe | NR | 138 |
| **17360809** | Chan | 2007 | Western Pacific | 2004 | 300 |
| **17539290** | Khadka | 2007 | South East Asia | NR | 29 |
| **17543399** | Cheng | 2007 | Western Pacific | NR | 70 |
| **17988957** | Zakerbostanabad | 2008 | Eastern Mediterranean | 2006 | 28 |
| **18006278** | Nusrath Unissa | 2008 | Western Pacific | NR | 80 |
| **18284842** | Martilla | 2008 | Europe | 2004 | 120 |
| **18445950** | Bostanabad | 2008 | Europe | 2005 | 46 |
| **18508939** | Abe | 2008 | Western Pacific | NR | 61 |
| **18573039** | Perdigao | 2008 | Europe | 2003 | 58 |
| **18653964** | Aslan | 2008 | Europe | 2006 | 35 |
| **18653966** | Guo | 2008 | Western Pacific | 2007 | 70 |
| **18687558** | Clemente | 2008 | Americas | 2003 | 20 |
| **19012077** | Ali | 2009 | Eastern Mediterranean | NR | 72 |
| **19090721** | Doustdar | 2008 | Eastern Mediterranean | 2006 | 73 |
| **19138546** | Abbadi | 2009 | Eastern Mediterranean | NR | 17 |
| **19146757** | Brossier | 2009 | Europe | 2004 | 113 |
| **19183459** | Abdelaal | 2009 | Eastern Mediterranean | NR | 26 |
| **19228426** | Dalla costa | 2009 | Americas | 2004 | 224 |
| **19250607** | Han | 2009 | Western Pacific | NR | 121 |
| **19520719** | Bolotin | 2009 | Americas | 2001 | 502 |
| **19547874** | Zenteno-Cuevas | 2009 | Americas | 2007 | 25 |
| **19581462** | Ho | 2009 | Western Pacific | NR | 142 |
| **19604720** | Cai | 2009 | WW | NR | 39 |
| **19628903** | Wang | 2009 | Western Pacific | 2008 | 45 |
| **19673965** | GarzaGonzales | 2010 | Americas | NR | 171 |
| **19710078** | Valvatne | 2009 | South East Asia | 2002 | 96 |
| **19741059** | Choi | 2010 | Western Pacific | 2006 | 76 |
| **19750330** | Siquira | 2009 | Americas | 2003 | 102 |
| **19846642** | Bravo | 2009 | Western Pacific | NR | 102 |
| **19861006** | Scaaf | 2009 | Africa | 2005 | 64 |
| **19893355** | Cho | 2009 | Western Pacific | 2008 | 141 |
| **20008778** | Luo | 2010 | Western Pacific | 2008 | 242 |
| **20074413** | Hu | 2010 | Western Pacific | 2005 | 351 |
| **20538518** | Homolka | 2010 | Africa | 2004 | 230 |
| **20708497** | Li | 2010 | Western Pacific | 2008 | 116 |
| **20713679** | Khanna | 2010 | South East Asia | NR | 6 |
| **21110864** | Ioerger | 2010 | Africa | NR | 14 |
| **21146714** | Siu | 2011 | Western Pacific | 2009 | 24 |
| **21244531** | Ando | 2011 | Western Pacific | 2008 | 74 |
| **21276306** | Al-mutairi | 2011 | Eastern Mediterranean | NR | 82 |
| **21300839** | Campbell | 2011 | WW | 2008 | 314 |
| **21396209** | Imperiale | 2011 | Americas | 2009 | 90 |
| **21514309** | Coelho | 2011 | Americas | 2004 | 41 |
| **21554227** | Bostanabad | 2011 | Eastern Mediterranean | 2008 | 46 |
| **21569121** | Nimri | 2011 | Eastern Mediterranean | 2007 | 6 |
| **21617314** | Kozhamkulov | 2011 | Europe | 2009 | 310 |
| **21717327** | Soudani | 2011 | Eastern Mediterranean | 2008 | 43 |
| **21911575** | Ali | 2011 | Eastern Mediterranean | 2009 | 50 |
| **22012218** | Alves | 2011 | Americas | 2007 | 15 |
| **22078903** | Yoon | 2012 | Western Pacific | 2008 | 80 |
| **22162548** | Perez-osorio | 2012 | Americas | NR | 114 |
| **22170905** | Minh | 2012 | Western Pacific | 2009 | 92 |
| **22325117** | Jin | 2012 | Western Pacific | 2009 | 237 |
| **22450970** | Poudel | 2012 | South East Asia | 2010 | 158 |
| **22461677** | Engstrom | 2012 | WW | NR | 215 |
| **22470121** | Fenner | 2012 | Europe | 2008 | 152 |
| **22507192** | Greif | 2012 | Americas | 2005 | 45 |
| **22553245** | Yuan | 2012 | Western Pacific | 2011 | 77 |
| **22646308** | Feuerriegel | 2012 | Africa | 2004 | 97 |
| **22708343** | Madania | 2012 | Eastern Mediterranean | 2010 | 72 |
| **22747769** | Yadav | 2012 | South East Asia | 2010 | 37 |
| **22792333** | Chia | 2012 | Americas | 2011 | 67 |
| **22863574** | Rahim | 2012 | South East Asia | 2010 | 217 |
| **22943573** | Ballif | 2012 | Western Pacific | 2010 | 27 |
| **22972833** | Daum | 2012 | Africa | 2011 | 26 |
| **23146281** | Poudel | 2013 | South East Asia | 2010 | 13 |
| **23317963** | Yadav | 2013 | South East Asia | 2010 | 44 |
| **23453008** | Escalante | 2013 | Americas | 2001 | 28 |
| **23539241** | Machado | 2013 | Europe | 2011 | 17 |
| **23561273** | Jnawali | 2013 | Western Pacific | 2010 | 190 |
| **23744165** | Jagielski | 2013 | Europe | 2004 | 46 |

*Only specimens with the specific location of the mutation identified, the specific amino acid and/or nucleotide change described, and phenotypic result noted were extracted from each manuscript.
